# Modeling predictors of incomplete antenatal care utilization among reproductive-age women in Ethiopia using machine learning algorithms and SHAP interpretation

**DOI:** 10.1371/journal.pdig.0001489

**Published:** 2026-07-09

**Authors:** Jibril Bashir Adem, Anas Ali Alhur, Abdene Weya Kaso, Meron Asmamaw Alemayehu, Shimels Derso Kebede, Agmasie Damtew Walle, Daniel Niguse Mamo, Ermias Bekele Enyew

**Affiliations:** 1 Department of Public Health, College of Health Sciences, Arsi University, Asella, Ethiopia‌‌; 2 Department of Health Informatics, College of Public Health and Health Informatics, University of Hail, Hail, Saudi Arabia; 3 Department of Epidemiology and Biostatistics, Institute of Public Health, College of Medicine and Health Sciences, University of Gondar, Gondar, Ethiopia; 4 Department of Health Informatics, School of Public Health, College of Medicine and Health Science, Wollo University, Dessie, Ethiopia; 5 Department of Health Informatics, School of Public Health, Asrat Woldeyes Health Science Campus, Debre Berhan University, Debre Birhan, Ethiopia; 6 Department of Health Informatics, School of Public Health, Arba Minch University, Arba Minch, Ethiopia‌‌; Mayo Clinic Arizona, UNITED STATES OF AMERICA

## Abstract

The incidence of maternal deaths from preventable pregnancy-related conditions remains alarmingly high at 303,000 annually, with over 800 women dying daily from avoidable causes. Ethiopia is one of eight sub-Saharan African countries that are identified as global hot spots for maternal mortality. Thus, this study aimed to model predictors of incomplete ANC utilization among reproductive-aged women in Ethiopia using explainable machine learning algorithms. This study employed the 2019 Ethiopian Mini Demographic and Health Survey (EMDHS) dataset. Data preparation techniques such as feature engineering, data splitting, handling missing values, resolving imbalanced categories, and outlier removal were used to clean the data. Six popular machine learning classifiers were implemented in R 4.4.2 and Python 3.11.5 via Jupyter Notebook through the Scikit-learn and XGBoost packages and evaluated using multiple permanence matrices. Finally, Shapley Additive exPlanations (SHAP) analysis was used to clarify the impact of the most important predictors on the model’s output. This study included 3979 women who had given birth during the five years prior to the survey out of the 8,885 interviewed women. Random forest (RF) was found to be the best model for modeling predictors of incomplete ANC utilization in Ethiopia, with 73% accuracy and 79% area under the ROC curve. Older age (25 and 34), residence area, being in the Benishangul-Gumuz, Tigray region, Harari region and wealth indices were top predictors of incomplete ANC utilization among reproductive-age women in Ethiopia. This study found that young women in rural areas, having low-income indices and low levels of education, as well as those living in the Somali and Harari regions, are more likely to experience incomplete ANC utilization. Policymakers and stakeholders should prioritize these vulnerable groups when designing policies and maternal health services to improve ANC utilization and reduce maternal mortality in Ethiopia.

## Introduction

Complications during pregnancy and childbirth are the major causes of death and disability among women of reproductive age worldwide [1]. The incidence of maternal deaths from preventable pregnancy-related conditions remains high at 303,000 per year, accounting for 66% of all maternal cases globally, with over 800 women dying every day from avoidable causes related to pregnancy and childbirth [2,3]. Among the estimated annual maternal deaths worldwide, approximately 86% occur in sub-Saharan Africa (SSA) countries and southern Asia, with SSA alone accounting for more than 62% [4]. Ethiopia is one of eight sub-Saharan African nations that have been classified as worldwide hotspots for maternal mortality [3]. In Ethiopia, 19,000 maternal fatalities occur annually, with a high maternal death rate of 412 per 100,000 live births [5,6]. Promoting health and preparing women and their families physically, emotionally, and psychologically for motherhood are essential steps throughout pregnancy to lower maternal and child mortality [7,8].

Antenatal care (ANC) is a maternal health service provided by trained medical professionals to pregnant women and adolescent girls to ensure the greatest possible health outcomes for the mother and the unborn child [8]. It is regarded as one of the most important factors for the mother’s health and for the optimal development of the fetus, as well as for preventing or reducing difficulties during pregnancy [9]. In an effort to guarantee that pregnant women receive proper treatment, the World Health Organization (WHO) initially advised that all pregnant women have at least four prenatal visits, which has now been revised to include up to eight visits during pregnancy [10,11].

Poor antenatal care visits, late appointments, or fewer visits than recommended have been found to be associated with poor pregnancy outcomes [12]. For example, a systematic review and meta-analysis on the effects of antenatal care on perinatal outcomes in Ethiopia revealed that a 38% decrease in perinatal death, a 66% decrease in still birth, and a 15% decrease in early neonatal death were strongly associated with better ANC visits among reproductive-aged women [13]. A cohort study carried out in Ethiopia also revealed that having completed ANC visits was significantly associated with reductions of 81.2%, 61.3%, 52.4%, and 46.5% in postpartum hemorrhage, early newborn death, preterm labor, and low birth weight, respectively [14]. Similarly, a study conducted on the effect of optimal antenatal care on maternal and perinatal health in Ethiopia revealed that optimal ANC had a positive effect on reducing perinatal death due to respiratory and cardiovascular disorders and extending intrauterine life by one week. The effects on maternal health include avoiding the risk of uterine rupture, improving the utilization of operative vaginal delivery and avoiding delays in the decision to seek care [15].

The magnitude of ANC utilization among reproductive-age women in Ethiopia ranged from the highest in Addis Ababa (82.7%) to the lowest in Somalia (11.3%) [16] and 12.0% in Dejen and Aneded Districts of Northwest Ethiopia [17]. Women in Addis Ababa had the lowest percentage (3.2%) of zero ANC visits, whereas those in Somalia had the highest percentage (70.8%) [16]. Socio-demographic and economic factors, such as maternal educational status, age, residence, wealth indices, occupation, and religion; clinical and health service-related factors, such as pregnancy status, number of children, access to health services, health insurance coverage, and pregnancy complications; and personal factors, such as lack of knowledge and a negative attitude toward maternal health services, smoking cigarettes, chewing khat, drinking alcohol, and having multiple sexual partners, have all been found to be determinants of ANC utilization in Ethiopia [18–20]. All of the studies on antenatal care utilization in Ethiopia, used conventional statistical methods and were conducted on a small scale at the local level, offering no previously unknown facts and leaving a gap in literature for machine learning algorithms to identify hidden patterns, relationships, and predicting the future status of this public health issue [21,22].

Machine learning is an advanced technique used in data science to sort, locate relevant data, and develop models for association, classification, and prediction [23]. These algorithms are important to extract complex relationship and hidden patterns from data that traditional statistical techniques like logistic regression overlook [24,25]. Numerous researches have been conducted using a range of machine learning algorithms to address various maternal health issues. For example, an analysis of trunk movement patterns in women with postpartum low back pain using machine learning revealed that the optimal Optuna regressors performed the best in terms of regression, with a mean squared error of 0.000273; mean absolute error of 0.0039, and R2 score of 0.9968. Both the Random Forest Classifier and the simple CNN outperformed other models in classification, achieving near-perfect 100%, accuracy, precision, recall, area under the receiver operating characteristic curve (AUC), and F1-score [26].

Similarly study conducted on the prediction of female pelvic tilt and lumbar angle using machine learning in case of urinary incontinence and sexual dysfunction found that best performing model for Pelvic tilt and Lumbar angle prediction were AdaBoost (R2 = 0.944) and decision tree (R2 of 0.976) [27]. Furthermore, a study on revolutionizing core muscle analysis in female sexual dysfunction using machine learning revealed that CNN and random forest regressors are the most accurate models for predicting changes in core muscles during FSD. CNN had the lowest MSE (0.002) and the highest R2 score (0.988), while random forest regressors fared well with an MSE of 0.0021 and an R2 score of 0.9905 [28].

To gain a more comprehensive understanding of ANC utilization and its determinants, this study employs six popular machine learning (ML) algorithms [29], namely, logistic regression (LR), random forest (RF), K-nearest neighbors (KNN), adaptive boosting (AdaBoost), naive Bayes (NB), and support vector machines (SVMs), all of which are binary classifiers, to identify complex patterns and interactions between variables. These algorithms are capable of handling large datasets and identifying complex patterns that conventional approaches can overlook. They are capable of learning from data without being explicitly programmed, allowing for more accurate predictions and insights. A SHapley Additive exPlanations (SHAP) [30] analysis was applied to identify the most influential predictors of ANC Use in Ethiopia. These advanced techniques provide a more nuanced understanding of ANC utilization and its determinants, enabling policymakers to make data-driven decisions and implement targeted interventions to improve maternal health outcomes.

## Methods

### Study design, setting and period

The study used community-based cross-sectional survey data from the 2019 Ethiopian Mini Demographic and Health Survey (EMDHS) dataset. These data were collected from nine regional states and two administrative cities (Addis Ababa and Dire-Dawa) in urban and rural areas of Ethiopia by the Central Statistical Agency (CSA) and Ethiopian Public Health Institute (EPHI) under the Ministry of Health’s supervision between March and June 2019 [31]. Ethiopia is one of the most popular nations in the Horn of Africa, with a population of over 112 million and a diverse landscape [32]. The country was divided into nine regions and two administrative cities during the study period: Gambela, Harari, Southern Nations Nationalities and People (SNNP), Oromia, Somalia, Benishangul-Gumuz, Afar, Amhara, Tigray, and Afar. Administratively, the region is divided into zones, which are further divided into Woredas, which are further divided into the smallest entity, Kebele.

### Data source

The 2019 EMDHS Women’s dataset (IR file) served as a data source for the analysis. The Woman Questionnaire was used to collect information from all eligible women of childbearing age (15–49 years), and the electronic data collection system was utilized in the 2019 EMDHS. The data were obtained from the measure DHS website, https://dhsprogram.com/data/available-datasets.cfm, and accessed upon reasonable request.

### Study population, sample size and procedure

All women who had a live birth within the five years before the survey were included in the study to reduce recall bias from time relapse, and women who had two or more live births were assessed on the basis of the most recent birth. Among the 9,012 eligible women in the investigated households, 8,885 participated in individual interviews. The study sample was chosen by stratified, two-stage cluster sampling. Using a probability proportional to the size of the EA, 305 enumeration areas (EA) in 93 urban regions and 212 rural areas were chosen. Using the newly constructed household listing, a fixed number of 30 households per cluster were chosen in the second stage of selection, with an equal chance of systematic selection.

### Study variables

#### Outcome variables.

In this study, antenatal care utilization was considered an outcome variable, which was dichotomized into complete (coded as 0) and incomplete follow-up (coded as 1). Pregnant women in Ethiopia who received four or more prenatal care visits were considered to have completed ANC follow-up, whereas those who received fewer visits were considered to have incomplete ANC follow-up [33,34].

#### Predictor variables.

Various socio-demographic, economic, maternal and health service-related factors were included as predictor variables. Socio-demographic and economic factors included mothers’ current age (categorized in to 15–24 years, 25–34 years and 35–49 years), region (included Gambela, Harari, Southern Nations Nationalities and People (SNNP), Oromia, Somalia, Benishangul-Gumuz, Afar, Amhara, Tigray, and Afar and two cities namely Addis Ababa and Dire Dawa), residence (urban and rural), religion (Christian, Muslim and Other which includes Wakefata and Atheist) and educational level (categorized as no schooling, primary education, and secondary and above), sex of the household head (Male and Female), and relationship (grouped in to head of the household, wife, daughter or daughter-in-law, other relatives and not related), family wealth indices (poorest, middle and richest), household size (grouped in to 1–3 members, 4–5 members, 6 and above members) and marital status (grouped in to never married, Married and widowed/divorced/separated).

Maternal and health service-related factors included Number of under five children (grouped in to no under_5 children and 1 and more under_5 children), number of children ever born (grouped in to 1–3 children and 4 and more children), birth in the last 5 years (1–2 birth and 3–5 birth), birth in the last year (no birth and 1 birth), knowledge about reproductive methods (knows about modern methods, knows about traditional methods only, and no knowledge about any method), cesarean delivery (no and yes) and age at first birth (below 15 years, between 16 and 34 years and above 35 years).

### Data processing and analysis

This study applied a supervised machine learning (ML) approach using the fundamental framework from previous studies, which is based on Yufeng Guo’s seven steps of machine learning, to predict ANC utilization and identify its key determinants among women of childbearing age in Ethiopia. [29,35]. The use of Yufeng Guo’s seven-step machine learning framework presents an innovative and empirically sound analysis strategy for highly complex survey such as 2019 Ethiopian Mini Demographic and Health Survey (EMDHS). This analysis strategy is crucial to account for the national representativeness of the data sample by applying sampling weights, preprocessing clustered and high-cardinality categorical variables, and utilizing robust ensemble algorithms with hyperparameter tuning and cross-validation to reduce the risk of overfitting and to capture any nonlinear interactions that may exist in the datasets due to the way the survey was designed (multi-stage cluster sampling; hierarchical structure of households, women, and children; and class imbalance across major health outcomes).

This framework will address contextually derived heterogeneity as well as the prevalence of non-random missingness (data not present) within the DHS datasets by utilizing KNN imputation and maintaining local similarities among socioeconomic and regional characteristics associated with the data. Furthermore, methods such as Boruta, SMOTE and SHAP approaches were used to further enhance both the interpretability and the predictive validity of a model, while also creating a methodological framework that was well aligned with the limitations and complexity of the EMDHS dataset. Variable extraction and imputation were conducted via R software version 4.4.3. Using Jupyter Notebook, the Scikit-learn machine learning (ML) algorithm was built in Python 3.11.5 [36–38].

### Data preprocessing

Data cleaning feature engineering, dimensionality reduction, and data splitting are among the techniques used for data preprocessing in this study.

#### Data cleaning.

Data cleaning procedures were performed after the data were extracted from the database, which included resolving imbalances in the outcome variables, imputing missing values, and identifying outliers. To address the missing values for independent variables of the dataset, the k-nearest neighbors (KNN) imputation method was applied due to its accuracy and sensitivity in preserving data structure [39]. This method was used as the dataset had a small percentage of missing data (8%), Little’s MCAR test showed that the missing data mechanism was Missing Completely at Random (MCAR) (p > 0.05), and the relationships between the variables were not strictly linear, which made KNN imputation more appropriate than parametric methods. Compared to more complex and/or sophisticated data imputation techniques such as multiple imputation, next generation data imputation, model-based data imputation, and approximated data management, KNN is not only less expensive and time-consuming to compute, but it also relies less on data assumptions, such as a normal distribution of cases, and is less likely to introduce model bias when working with limited missing data. KNN is a practical, reliable, and methodologically appropriate way to impute missing values, particularly in our scenario, because of the relatively small amount of MCAR data available and the requirement to maintain/match the original structural integrity of the data set in most cases [40,41].

#### Feature engineering and dimensionality reduction.

The raw data were transformed into features that better reflect the underlying problem for predictive models to improve model accuracy on unobserved data [42]. Since all model input features were originally categorical, the one-hot encoding technique was implemented via the Pandas get_dummies () method which involve generating a new binary (0 or 1) feature for each distinct category in the original categorical variable, enabling the machine learning algorithm to treat each category as a separate entity [43]. Next, Recursive Feature Elimination with Cross-Validation (RFECV) was used to efficiently find the most significant characteristics even with a limited feature set, while also accounting for any interactions between variables via its iterative approach. This is useful in instances where a few features have complex interactions with the target variable [44]. While dimensionality-reduction methods such as Principal Component Analysis (PCA) are useful for continuous data, they are not appropriate for one-hot-encoded categorical features. As a result, to ensure that only the most informative dummy variables were included, we merely reduced the number of features using RFECV.

### Model selection

Appropriate models were chosen for training after the data were prepared and divided into training and testing sets. Given the categorical nature of the outcome variable, which was categorized into complete and incomplete ANC utilization, binary classifiers were employed for both training and testing the data. The strongest predictors of ANC utilization among Ethiopian women of reproductive age were thus identified by comparing six popular machine learning (ML) algorithms: logistic regression (LR), random forest (RF), K-nearest neighbors (KNN), adaptive boosting (AdaBoost), naive Bayes (NB), and support vector machines (SVMs) [45]. These models were selected to provide comprehensive comparisons across various model types, such as instance-based learning, ensemble approaches, probabilistic classifiers, and linear models.

**Random forest (RF)** is a supervised machine learning algorithm that combines the outputs of several decision trees to create a more accurate prediction. Because of the random sampling of data and features, each decision tree in the “forest” is slightly different, making the model more robust and having less over-fitting. It can be applied to both classification and regression tasks [46].

**K-nearest neighbors (KNN)** is a nonparametric supervised learning classifier that uses closeness to predict or classify the grouping of individual data points. It is currently one of the most popular and simple classification and regression classifiers in machine learning. Although it can be used for regression or classification problems, the KNN algorithm is typically used as a classification tool because it is based on the assumption that similar points can be found near one another [47].

**Adaptive boosting (AdaBoost)** is a machine learning algorithm that iteratively changes weights on training data points and focuses more on the misclassified instances in each round to create a single, more powerful “strong learner” by combining multiple “weak learners” (classifiers that perform only marginally better than random guessing). The classifier is strengthened, and the overall classification accuracy is increased by gradually correcting the mistakes made by previous weak learners [48].

**Logistic regression (LR)** is a supervised learning technique that examines the relationships between independent factors and binary outcome variables. It is most appropriate for binary classification tasks. It uses the sigmoid function to generate a value between 0 and 1 that expresses the probability of a given outcome (such as “yes” or “no”) on the basis of input data [49].

**The naive Bayes (NB)** algorithm is a supervised machine learning classification method that employs Bayes’ theorem to estimate the likelihood of a specific class label on the basis of provided data, assuming that all features are independent of one another. It is a simple yet effective tool for classification tasks, particularly when working with large datasets; text classification, spam filtering, and medical diagnosis are among its many applications due to its speed and ease of use [49].

**The support vector machine (SVM)** is a supervised machine learning algorithm that is particularly helpful for binary classification tasks. It separates data points that belong to different categories by determining the optimal hyper-plane (decision boundary) that maximizes the margin between different data classes. It can also convert data into higher-dimensional spaces via kernel functions, which handle both linear and nonlinear data [50].

### Balancing, model training and evaluation

Machine learning relies heavily on SMOTE for addressing class imbalance in datasets by generating synthetic data points for minorities. By using underrepresented data more effectively, models can learn more effectively, which improves the overall prediction accuracy, especially for instances of minority classes, particularly in scenarios where correctly identifying the minority class is important. Therefore, to balance the unequal distribution of the outcome variable, SMOTE oversampling was used in this study to provide additional synthetic observations from the minority category after the total dataset (3979) were split into (80%) training and (20%) test sets.

The selected model was trained on prepared data following the model selection process, and then tenfold cross-validation was used to compare the models’ performances. Following comparison, the best predictive model was selected, and it was trained on balanced training data to provide the final prediction on unseen test data [29]. Performance metrics, including overall accuracy, precision, recall, and F1 score, are computed in this study via confusion measures to evaluate the effectiveness of the selected classifiers. Additionally, the model performance was assessed via receiver operating characteristic (ROC) curves.

### Hyper-parameter tuning

In machine learning, a “hyper parameter” is a configuration setting that supervises a model’s learning process but is not learned directly from the data during training; rather, it must be manually set by the user prior to training. By affecting factors such as complexity and training speed, a “hyper parameter” can have a significant effect on the model’s performance; in other words, it is a parameter that controls how the model learns rather than what it learns from the data [51]. Thus, Optuna was used to tune the hyper-parameters of the best model after model selection. Using Optuna, hyper-parameter optimization is formulated as the process of minimizing or maximizing an objective function (such as accuracy) by using a set of hyper-parameters as inputs. By using the Bayesian framework, we can better understand the probability of the optimal values and avoid unnecessary computation when combining non-performing parameters [52]. This framework is used as it is more efficient and flexible than grid search and randomized search, which take explicitly user-defined hyper-parameters and optimize the model only by those hyper-parameters.

### Prediction, model interpretation and explanation

**Fig 1:**
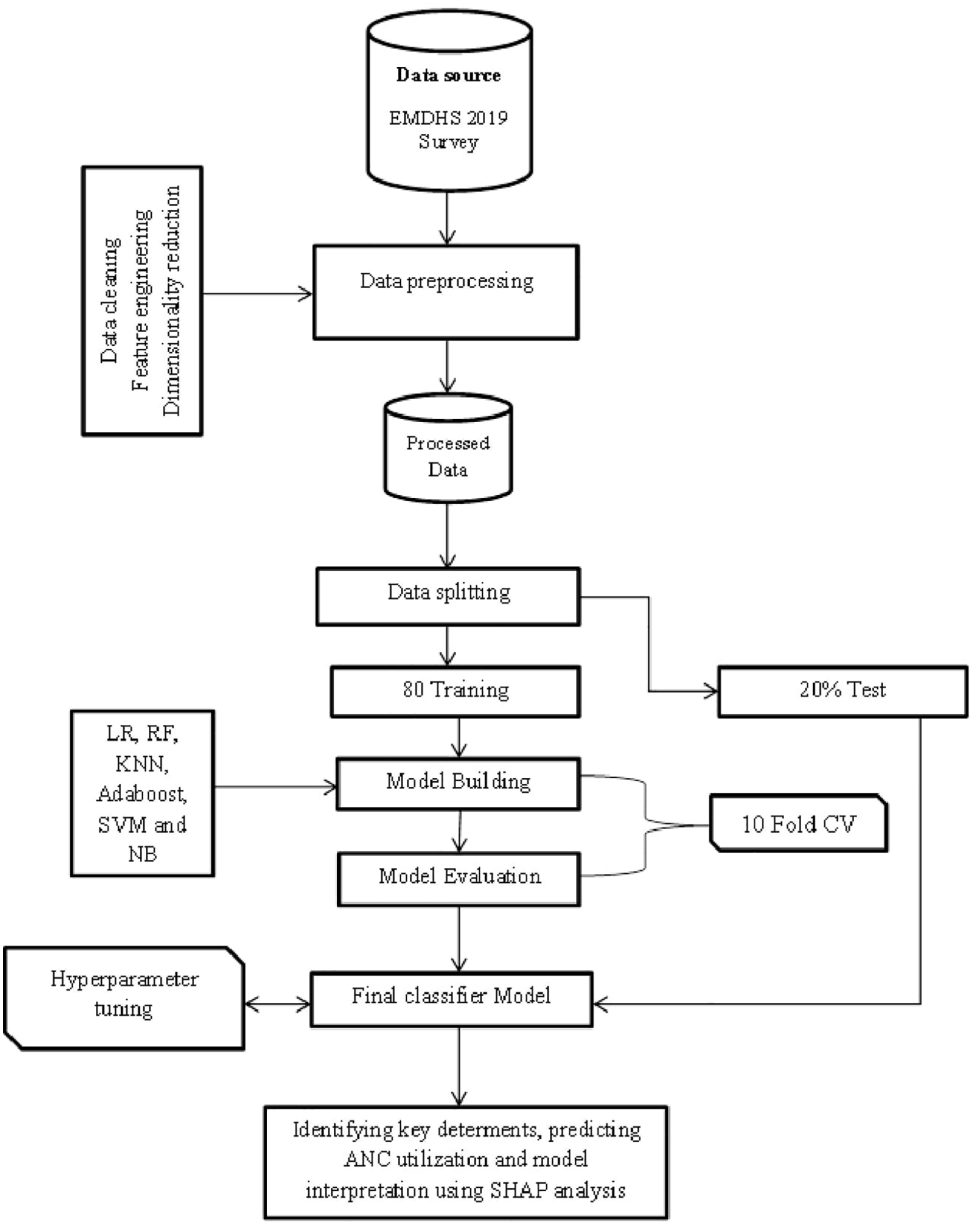
Data preparation and analysis plan flowchart.

ANC utilization among Ethiopian women of reproductive age was predicted in this study via the best predictors, which were selected by the best-performing classifier on the basis of a variety of parameters, including accuracy, precision, recall, AUC, and F1 score. To analyze and explain the model output, a machine learning framework called SHAPs was employed. An integrated method for understanding the importance of input features for prediction is offered by the Shapley values concept, which originated from cooperative game theory [53]. It indicates the degree to which the value of each feature varies from its mean contribution to the predictions when paired with different values of other features [54]. Finally, the contribution of each component to the prediction of a positive class was demonstrated via a waterfall plot (**Fig 1**).

### Data quality assurance

The study employed the most recent national survey, which was conducted by esteemed organizations, including Ethiopia’s Central Statistical Agency (CSA) and Ethiopian Public Health Institute (EPHI), under the supervision of the Ministry of Health. To ensure that data quality was missing, categorical variables were encoded, numerical features were normalized, and outliers were handled throughout the data cleaning process. The SMOTE was used to address class imbalance, and tenfold cross-validation and data consistency checks were employed to improve robustness and integrity. Machine learning relies heavily on SMOTE for addressing class imbalance in datasets by generating synthetic data points for minorities. By using underrepresented data more effectively, models can learn more effectively, which improves the overall prediction accuracy, especially for instances of minority classes, particularly in scenarios where correctly identifying the minority class is important.

### Ethics approval and consent to participate

The study adhered to the principles of the declaration of Helsinki and the ethical review committee of Arsi University’s College of Health Sciences ruled that no formal ethical approval and informed consent was required in the study as it was based on publicly accessible data sources. The data used for analysis were available in the public domain through the measure DHS website (https://dhsprogram.com/data/available-datasets.cfm), and permission to use the data were granted by the measure DHS website through legal registration and reasonable requests.

## Results

### Socio-demographic and economic characteristics of the study participants

This study included 3979 women who had given birth during the five years prior to the survey out of the 8,885 women who were interviewed. With a mean age of 27.5 years and a standard deviation of ±9.1 years, more than half (51.1%) of the study participants were in the 25–34 years age range. The majorities of the study participants (74.77%) were from rural areas and were from Oromia (12.34%), SNNPR (11.79%), or the Amhara region (10.25%). The majority of women had no formal education (51.9%), were married (92.64%), and were Christian (52%) (Table 1).

**Table 1:** Socio-demographic and economic characteristics of the study participants, evidence from the EMDHS 2019 survey.

Variables	Group	Frequency	Percentage (%)
Age	15 - 24 years	1054	26.49
	25 - 34 years	2034	51.12
	35 - 49 years	891	22.39
Region	Tigray	346	8.69
	Afar	389	9.78
	Amhara	408	10.25
	Oromia	491	12.34
	Somali	342	8.59
	Benishangul	371	9.32
	SNNPR	469	11.79
	Gambela	338	8.49
	Harari	307	7.72
	Addis Ababa	236	5.93
	Dire Dawa	282	7.09
Residence	Urban	1004	25.23
	Rural	2975	74.77
Education level	No formal Education	2065	51.90
	Primary Education	1306	32.82
	Secondary and above	608	15.28
Religion	Christian	2069	52.00
	Muslim	1862	46.80
	Other*	48	1.21
Relationship to household head	Head of the household	620	15.58
	Wife	2915	73.26
	daughter or daughter-in-law	326	8.19
	other relatives	89	2.24
	Not related	29	0.73
Sex household head	Male	3164	79.52
	Female	815	20.48
Age household head	15 to 24 years	251	6.31
	25 to 34 years	1438	36.14
	35 to 49 years	1664	41.82
	Above 50 years	626	15.73
Household size	1 to 3 members	580	14.58
	4 to 5 members	1340	33.68
	6 and above members	2059	51.75
Marital status	Never married	29	0.73
	Married	3686	92.64
	widowed/divorced/separated	264	6.63
Wealth indices	Poorest	1872	47.05
	Middle	586	14.73
	Richest	1521	38.23

Note that other* includes traditional and atheist.

### Maternal characteristics of the study participants

With respect to the maternal characteristics of the study participants, the majority (95.43%) had one or more children under the age of five, more than half had one to three children, the majority (68.66%) had not given birth in the past year, and almost 7% had a cesarean delivery (**Table 2**).

**Table 2:** Maternal characteristics of the study participants.

Variables	Category	Frequency	Percentage (%)
Number of under_5 children	No under_5children	182	4.57
	1 and more under_5 children	3797	95.43
Number of children ever born	1- 3 children	2168	54.49
	4 and more children	1811	45.51
Birth in last 5 years	1 - 2 birth	3754	94.35
	3 - 5 birth	225	5.65
Birth in last year	No birth	2732	68.66
	1 birth	1228	30.86
	More than 1 birth	19	0.48
Knowledge about reproductive method	Knows about modern method	3706	93.14
	Knows about traditional method only	8	0.20
	No knowledge about any method	265	6.66
Cesarean delivery	No	3708	93.19
	Yes	271	6.81
Age at First Birth	Below 15 years	906	22.77
	between 16 and 34 years	3058	76.85
	Above 35 years	15	0.38

### The results of machine learning analysis

#### Feature selection.

**Fig 2:**
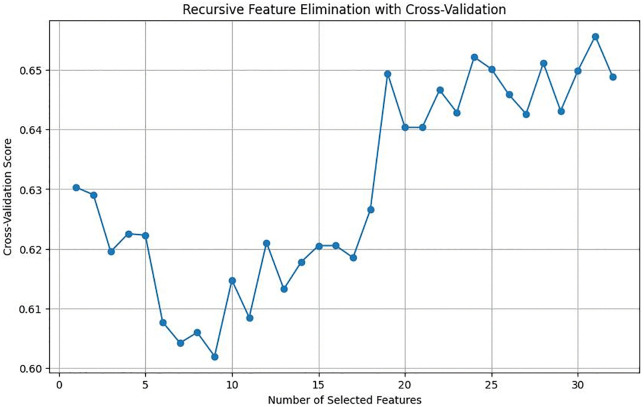
Recursive feature elimination with 10-fold cross-validation for feature selection.

To select the most important features in this study, recursive feature elimination with a 10_fold cross validation (RFECV) strategy was used for feature selection, as illustrated in Fig 2. The graph indicates that the ideal number of features for this specific dataset and model is between 18 and 33 features, as this is where the cross-validation score peaks (**Fig 2**).

### Balancing data and model performance comparison

To balance the unequal distribution of the outcome variable, SMOTE oversampling was used in this study to provide 667 additional synthetic observations from the minority category of women with incomplete ANC follow-up after the dataset were split into (80%) training and (20%) test sets. The distribution of total ANC utilization status for each class was rebalanced from 1656 incomplete ANC follow-ups and 2323 complete ANC follow-ups to 2323 in each category to produce symmetric distributions for both groups to enable the development of accurate prediction models.

The classifiers on the unbalanced training data were evaluated via stratified 10-fold cross-validation, and the support vector machine was significantly performed best, with an accuracy of 69%, 75% area under the ROC curve, 68% precision, 66% recall, and 66% F1_score. After the training data were balanced via the SMOTE oversampling technique, a machine learning model comparison was performed to avoid the creation of a biased model. With 73% accuracy, 79% area under the ROC curve, 71% precision, 70% recall, and 70.5% F1 score, the random forest model was shown to be significantly reliable prediction model for ANC utilization among Ethiopian women of reproductive age. The performance of the models on balanced and unbalanced data is shown in **Table 3**.

**Table 3:** Model performance test statistics.

Models	Sampling	F1 Score	Accuracy	ROC-AUC	Recall	Precision
Logistic Regression **(reference)**	Unbalanced	0.65	0.68	0.74	0.66	0.68
	Balanced	0.70	0.71	0.78	0.70	0.70
Random Forest Classifier	Unbalanced	0.64(p ≤ 0.05)	0.65(p ≤ 0.01)	0.68(p ≤ 0.05)	0.64(p ≤ 0.05)	0.65(p ≥ 0.05)
	Balanced	0.705*(p ≤ 0.01)	0.73*(p ≤ 0.01)	0.79*(p ≤ 0.05)	0.70*(p ≤ 0.05)	0.71*(p ≤ 0.01)
Support vector machine	Unbalanced	0.66(p ≤ 0.01)	0.69(p ≤ 0.01)	0.75(p ≤ 0.05)	0.66(p ≤ 0.05)	0.68(p ≤ 0.05)
	Balanced	0.69(p ≤ 0.01)	0.70(p ≤ 0.05)	0.77(p ≥ 0.05)	0.70(p ≤ 0.05)	0.69(p ≤ 0.05)
k-nearest neighbors Classifier	Unbalanced	0.63(p ≥ 0.05)	0.65(p ≥ 0.05)	0.67(p ≤ 0.05)	0.63(p ≤ 0.05)	0.64(p ≥ 0.05)
	Balanced	0.68(p ≤ 0.05)	0.69(p ≤ 0.05)	0.75(p ≤ 0.05)	0.69(p ≤ 0.05)	0.70(p ≤ 0.05)
AdaBoost Classifier	Unbalanced	0.64(p ≥ 0.05)	0.66(p ≥ 0.05)	0.72(p ≥ 0.05)	0.65(p ≥ 0.05)	0.66(p ≥ 0.05)
	Balanced	0.68(p ≥ 0.05)	0.69(p ≤ 0.05)	0.76(p ≤ 0.05)	0.68(p ≤ 0.05)	0.69(p ≤ 0.05)
BernoulliNB	Unbalanced	0.62(p ≤ 0.05)	0.63(p ≤ 0.05)	0.73(p ≥ 0.05)	0.65(p ≥ 0.05)	0.65(p ≤ 0.05)
	Balanced	0.63(p ≤ 0.01)	0.64(p ≤ 0.05)	0.74(p ≤ 0.01)	0.64(p ≤ 0.05)	0.66(p ≤ 0.05)

### Hyper-parameter tuning of the random forest

Scikit-learn offer default hyper-parameters for all models, including random forest; however, it might not always lead to the optimal outcome. The performance of the random forests was optimized by changing the hyper-parameters with 100 trials on a particular search space via stratified 10-fold cross-validation. As a result, the maximum number of features that each tree considers when splitting a node (Max_features), the minimum number of samples needed for a leaf node (Min_samples_leaf), the number of decision trees in the forest (n_estimators), and the maximum number of samples each tree must be trained from independent variables (Max_samples) were used to optimize the model (**Table 4**).

**Table 4:** Hyperparameter tuning of the random forest model.

Hyper parameters	Default	Optimal value
Number of trees	100	250
max_features	square of the quantity of characteristics	0.41
Min_samples_leaf	2	4
n_estimators	1	2
Max_samples	None	0.79

### Prediction of ANC utilization among reproductive-aged women in Ethiopia

**Fig 3:**
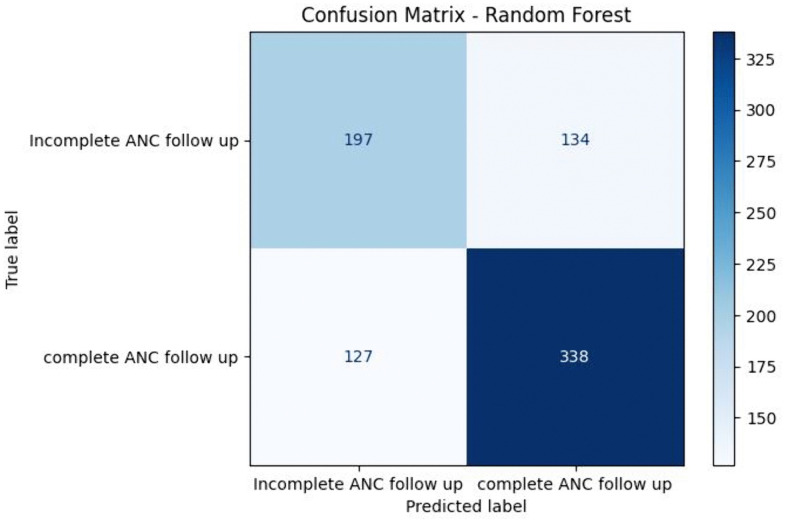
Confusion matrix of random forest model prediction on the test dataset.

The performance of the random forest model was evaluated via 778 (20%) test samples after the best model hyper-parameters were refined through training. The model accurately predicted 197 (true positive) out of 331 incomplete ANC utilizations and 338 (true negative) out of 465 complete ANC utilizations. However, as shown in Fig 5, the model mistakenly classified 127 complete ANC utilizations as incomplete (false negatives) and 134 incomplete ANC utilizations as complete (false positives). The model’s overall predictions on the test data were 68.8% accuracy, 72.7% recall, 71.6% precision and 72.2% F1 score (**Fig 3**).

**Fig 4:**
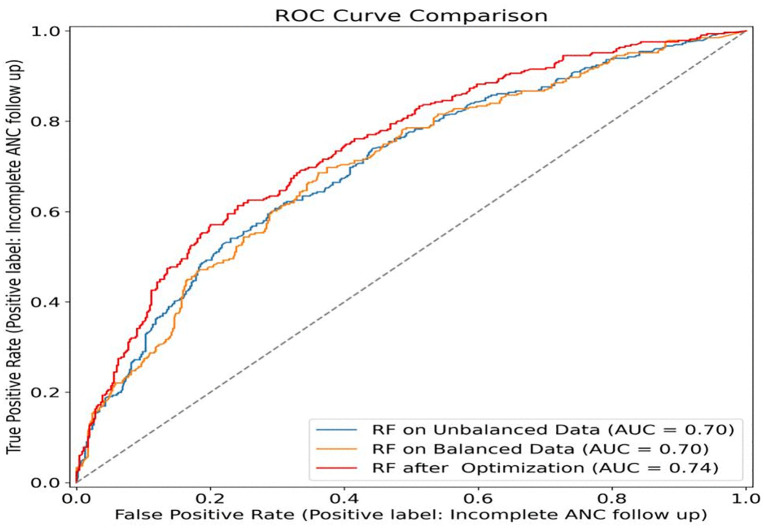
Comparison of the random forest model predictions on the test dataset.

The model’s performance on test data across different classification thresholds was also summarized via the ROC curve, which illustrates how sensitivity and specificity change. This makes it simple to define optimal thresholds to lower misclassification errors by providing a better understanding of the trade-offs between true and false positive rates. For unbalanced data, the random forest model achieved an AUC of 70%. After balancing and hyper-parameter tweaking, the prediction on the test data yielded AUCs of 70% and 74%, respectively, suggesting a strong predictive model (**Fig 4**).

### SHAP global feature importance to identify top predictors

To address the challenge of machine learning models with difficult-to-understand and opaque decision-making processes, this study used model-agnostic SHAPs version 0.45.0 to identify the most important predictors of ANC utilization among Ethiopian women of reproductive age and improve interpretation of the results. This approach investigates the mean absolute SHAP value for each predictor across all the data to quantify the contribution of each attribute to the expected ANC utilization status.

**Fig 5:**
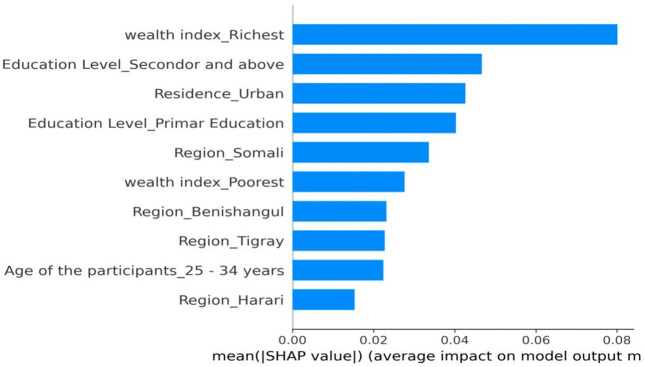
SHAP global feature importance plot of the optimized random forest model.

To predict the outcome variable, the features are ranked in descending order of importance, with qualities displaying higher mean absolute SHAP values considered more important. Therefore, the top predictors of ANC utilization among reproductive-aged women in Ethiopia were the richest wealth indices, secondary and above education level, urban residence, primary education level, being from the Somali region, poorest wealth indices, being from the Benishangul-Gumuz region, being from the Tigray region, being aged between 25 and 34 years and being from the Harari region (**Fig 5**).

### Model interpretation/explanation

#### SHAP Global interpretation.

**Fig 6:**
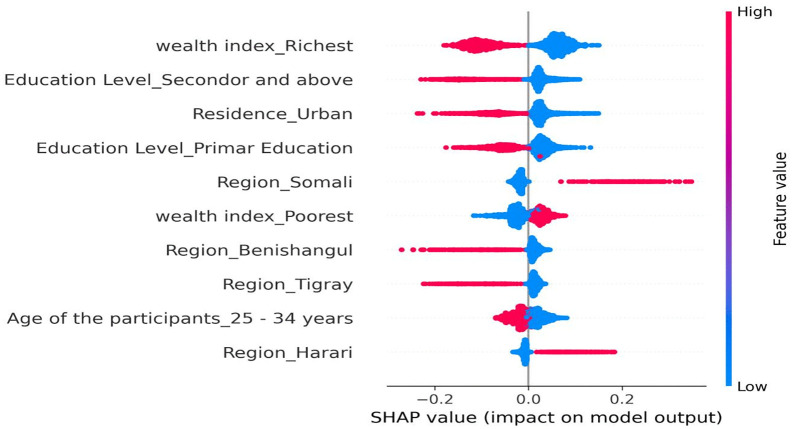
Beeswarm plot, ranked by the mean absolute SHAP value generated by the optimized random forest model.

A beeswarm plot was employed to analyze the derived feature importance, offering an in-depth explanation of the ways in which the variables affected the model’s predictions by plotting each predictor’s Shapley value for each sample to show the spread of their impact on the prediction of ANC utilization status. The importance and correlation of each of the top ten features on the outcome variable are indicated by the points on this beeswarm plot, which represent the Shapley values of the features associated with ANC utilization (**Fig 6**).

The figure’s red and blue colors correspond to each predictor’s higher and lower values, so the probability of incomplete ANC utilization is higher at points on the right side of the vertical line (0 SHAP value) and lower at points on the left. Accordingly, the likelihood of incomplete ANC utilization was lower for people who lived in urban areas, were between the ages of 25 and 34, lived in the Benishangul-Gumuz or Tigray regions, had high wealth indices, and had the highest level of education. However, the likelihood of incomplete ANC utilization was greater among participants from the Harari or Somali regions and those with the lowest wealth indices.

#### SHAP local interpretation.

**Fig 7:**
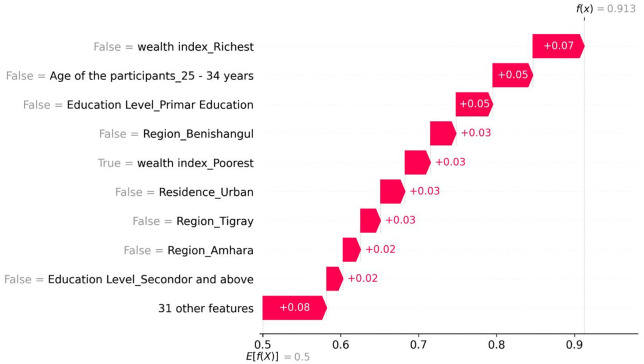
Waterfall plot displaying the prediction of the first observation.

**Fig 8:**
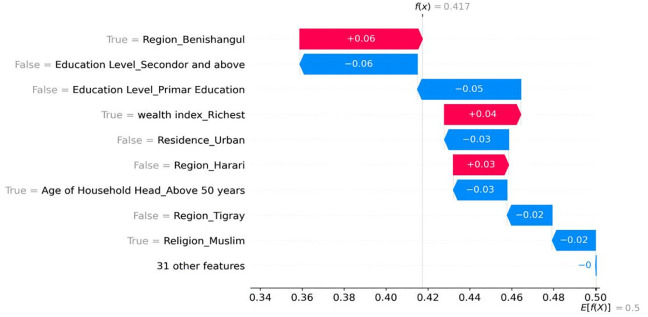
Waterfall plot displaying the prediction of the second observation.

A waterfall plot was used to interpret the model prediction and Figs 7 and 8 display the interpretations of the first and second observations. The waterfall plot in Fig 7 begins with the expected value of the model output on the x-axis (E[f(X)] = 0.5), which is the starting value predicted for the sample in question before accounting for any feature contributions. For the selected observation, a model output above (E[f(X)]) indicates a positive class (incomplete ANC utilization), whereas scores below this value indicate a negative class (complete ANC utilization). As a result, for the first observation, the predicted value output is moved to the final model output (f(x) = 0.913), which is categorized as the positive class by the combination of positive contributions (in red) and negative contributions (in blue). Therefore, the likelihood of incomplete ANC utilization for this particular woman was higher given that she was not in a highest wealth index, was not between the ages of 25 and 34, did not have a primary, secondary, or higher education, did not live in an urban area, or was not from the Benishangul–Gumuz, Amhara, or Tigray region (Fig 7).

Similarly, for the second observation, the combination of the positive contributions and the negative contributions moves the expected value output (E[f(x)] = 0.5) to the final model output (f(x) = 0.417). Therefore, there is a 58.3% (1.0–0.417) chance that this observation is not incomplete ANC utilization. Therefore, for this specific participant, the reasons that she did not attend primary, secondary, or higher education, did not reside in an urban area, had a household head who was older than fifty, was not from the Tigray region, and was not Muslim reduced the likelihood of incomplete ANC utilization. However, the participants’ likelihood of using ANC insufficiently increased given that she resided in Benishangul-Gumuz, were not from the Harari region, and had the highest wealth indices (Fig 8).

## Discussion

This study aims to predict ANC utilization and identify its key determinants among reproductive-age women in Ethiopia via machine learning models using the most current dataset from 2019 EMDHS applying SHAP analysis for global and local explanations of the outcomes. To achieve this goal, six machine learning classifiers were trained via tenfold cross-validation on both balanced and imbalanced training data. The conventional 80/20 split technique, in which 20% of the data were used for model testing and 80% for training was adopted for high-dimensional data [30]. The performance of the six classifier models was compared in terms of classification accuracy, F1 score, precision, recall, and AUC score. Accordingly, with an accuracy of 69%, 75% area under the ROC curve, 68% precision, 66% recall, and 66% F1_score, the support vector machine achieves the best results on unbalanced training data. To prevent the development of a biased model, a machine learning model comparison was conducted after the training data were balanced via the SMOTE oversampling technique, and the random forest was thus found to be the most accurate prediction model for ANC utilization among Ethiopian women of reproductive age, with 73% accuracy, a 79% area under the ROC curve, 71% precision, 70% recall, and a 70.5% F1 score.

Similarly, random forest has shown the best predictive performance in the study on machine learning modeling for identifying predictors of the unmet need for family planning among married/in-union women in Ethiopia [45], study on performance evaluation and comparative analysis of different machine learning algorithms in predicting postnatal care utilization [55], study on explainable machine learning algorithms to identify predictors of intention to use family planning among women of reproductive-age in Ethiopia [56] and on the prediction of contraceptive discontinuation among reproductive-aged women in Ethiopia via the Ethiopian Demographic and Health Survey 2016 dataset‌‌ [29]. However, studies conducted on the use of machine learning algorithms to predict unintended pregnancy [57] and the use of machine learning algorithms to predict modern contraceptive use in Ethiopian women of reproductive age [58] have revealed that the extra tree classifier and extreme gradient boosting (XGB) have the best predictive performance. These inconsistencies could be attributed to the size of the dataset, the number of features and the data type used in model development.

The SHAP analysis based on the RF model revealed that the risk of incomplete ANC utilization was lower among women who lived in urban areas, which is supported by the findings of other similar studies conducted in Nepal [59], Nigeria [60], Butajira town in southern Ethiopia [61], the Metekel Zone, northwestern Ethiopia [62] and studies conducted on the factors associated with antenatal care utilization in Ethiopia using the 2019 EMDHS dataset [8]. A possible explanation for this variation may be a lack of enough transportation, lower socioeconomic status, poor infrastructure, limited access to healthcare facilities, and a lack of awareness regarding the significance of antenatal care in rural areas [62,63].

The participants from the Harari or Somali regions had a greater likelihood of incomplete ANC utilization in this study than did those from the Benishangul-Gumuz and Tigray regions. The results of a study on the factors influencing the use of prenatal care in Ethiopia supported these findings, showing that women from the Somalia region were less likely than those from Tigray for complete ANC utilization [8]. Another study in Ethiopia reported that there are differences in ANC utilization around the country. The Tigray, Amhara, and Benishangul Gumuz regions, as well as the Addis Ababa and Dire Dawa city administrations, have the lowest rates of incomplete ANC utilization, whereas the Somali, Afar, and Gambela regions have the highest rates of incomplete ANC utilization [64]. This is may be due to the fact that, these regions have lowland populations primarily consisting of pastoralists and Agro-pastoralists. These communities tend to migrate through seasonal patterns in search of water and grazing land for their animals. Because Ethiopia’s health care system is mainly static and does not serve the mobile populations, it often cannot meet the needs of these highly mobile populations [65]. The low population density, spatial mobility, dispersion, and distance from health care services make it difficult for them to access health care services [66]. Religious and cultural beliefs, a lack of knowledge about ANC, a lack of husband support, a lack of funding for accommodation and transportation, and the absence of complete ANC service packages due to most health facilities’ shortages of personnel and supplies are some of the reasons for the low number of ANC visits in this regions [16,67]. For instance, a qualitative study on the barriers to reproductive, maternal, child, and neonatal health-seeking behaviors in Ethiopia discovered that in Somalia and other Muslim majority regions, except for a few mothers who have no specific sex preference, almost all mothers preferred to receive maternal health care services from female health care providers. Mothers who were served by a male attendant would not be satisfied with the services she received, and she may not visit the facility again for maternity services, as well as encourage others not to go to the institution [68]. This indicates the urgency of tailored policy and guidelines for maternal health service delivery in this area.

In this study, the risk of incomplete ANC utilization was lower for participants aged between 25 and 34 years; this finding is supported by the results of a systematic review and meta-analysis conducted among reproductive-aged women in Cameroon [69], an analysis of the Sub-Saharan African [70] and East African countries’ DHS datasets [71], a report of community-based panel studies on the extent of antenatal care received in Ethiopia [72] and a study on the regional disparities in antenatal care utilization among pregnant women and its determinants in Ethiopia [16]. Possible explanations for the age-based variation in ANC utilization include birth-related difficulties and deteriorating health as women age, which may lead them to seek more visits. Furthermore, compared with older women, young women (15–19 years old) are likely to lack experience with pregnancy care. This could be because women are more prone to use healthcare services as their knowledge and experience related to pregnancy increase [63,73].

The study also revealed that women with the highest level of education had a lower risk of incomplete ANC utilization. These findings are supported by studies conducted in Mauritania [74], Cameron [69], Ghana [75], and Sub-Saharan Africa [70]; a systematic review; a meta-analysis conducted in Ethiopia [76]; a study on the prevalence and factors associated with antenatal care utilization in Ethiopia using the 2016 EDHS dataset [77]; and a study on antenatal care utilization and associated factors among pregnant women in Ethiopia. A zero-inflated Poisson regression of the 2019 EMDHS dataset [78] revealed a negative correlation between women’s educational status and maternal health service utilization. This can be explained by women making better healthcare decisions and being more aware of the consequences of not using ANC services if they have greater education. Additionally, women who have more education are more knowledgeable about obstetric problems, which motivate them to use ANC services. Education enables women to hear health-related messages and gives them the skills and knowledge needed to obtain information. It also makes it easier for them to communicate with medical professionals [79,80]. These findings highlight the need for targeted maternal health education that engages religious leaders, community figures, and partners, with an emphasis on reaching women with lower levels of education.

This study revealed that the risk of incomplete ANC utilization was lower among women with a high wealth indices; this result is supported by the findings of a study conducted in rural Belgaum, Karnataka, India [81], in the pre-urban area of Aligarh [82], Nigeria [83], the 2016 EDHS dataset analysis on the prevalence and factors associated with antenatal care utilization in Ethiopia [77] and the result of zero-inflated Poisson regression of the 2019 EMDHS dataset on the number of antenatal care utilizations and associated factors among pregnant women in Ethiopia [45], which revealed that the highest wealth quintile was strongly associated with better ANC utilization. An explanation for the association between wealth quintiles and ANC utilization could be that antenatal care has both direct and indirect costs. In developing countries such as Ethiopia, where there is an enormous gap in health service access, even though antenatal care is provided for free, pregnant women are charged for transportation and other unnecessary costs when traveling to distant health institutions, which could delay women’s participation in early antenatal care and subsequent visits to the clinic. Indirect costs might therefore pose a barrier, although the services are provided freely [77,84].

### Strengths and limitations

The study was based on a 2019 EMDHS community-based survey; therefore, it did not explore clinical or health institution-related variables of antenatal care utilization and may not reflect the current situation. Similarly, the use of SHAP explanation may lead to misleading result due to its sensitivity to correlated variables, computational intractability of Shapley-value-based feature importance methods and reflection of internal model biases rather than true feature relevance, and a need to use complex workarounds like causal reasoning. Additionally, the lack of a similar study using a related approach (machine learning and SHAP analysis) impedes our writing in terms of comparison and rationale for our findings. Furthermore, due to the use of secondary data, the study may have missed significant variables such as behavioral factors, clinical and health service-related features, husband/partner-related factors, prior health service exposure, and paternal characteristics. Furthermore, because the data were gathered from women on the basis of their most recent birth history, the results might be susceptible to recall bias. In addition, the lack of a coefficient or odds ratio in the machine learning model’s output makes it difficult to determine the relative contributions of each variable to the final model.

However, an analysis of a nationally representative and comprehensive dataset utilizing supervised machine learning algorithms such as LR, RF, KNN, AdaBoost, NB, and SVM strengthened the validity and reliability of the study’s findings. To solve the problem with the black-box nature of machine learning models, information regarding each feature’s relative importance and contribution to the model’s predictions is provided by SHAP analysis. By characterizing complex model behavior, SHAP for both global and local feature importance helps users better comprehend and accept the model’s conclusions. This thorough methodology guarantees a deep understanding of the determining factors of incomplete ANC utilization among reproductive-aged women in Ethiopia.

## Conclusions and recommendations

This study employed six machine learning models to predict incomplete ANC utilization among reproductive-aged women and its determinants in Ethiopia. To prevent the development of a biased model, a machine learning model comparison was conducted using classification accuracy, the F1 score, precision, recall, and the AUC score after the training data were balanced via the SMOTE oversampling technique, and the random forest model was thus found to be the most accurate model for the prediction of incomplete ANC utilization and determinants among reproductive-aged women in Ethiopia, with 73% accuracy, a 79% area under the ROC curve, 71% precision, 70% recall, and a 70.5% F1 score.

According to the RF model-based SHAP analysis, women who were between the ages of 25 and 34, resided in an urban area, were from the Benishangul-Gumuz or Tigray regions, had a high-income index, and had the greatest level of education were less likely to have incomplete ANC utilization. However, those with the lowest wealth indices and those from the Somali or Harari regions were more likely to have incomplete ANC utilization. To improve ANC utilization among reproductive-age women in Ethiopia, alternative initiatives such as community outreach programs, mobile health services, or targeted subsidies for women living in rural areas, with low socioeconomic status, limited education levels, and from pastoralist and agro-pastoralist areas should be prioritized during policy formulation, decision-making, and health service provision.

## Supporting information

S1 File Full dataset analyzed to generate the results reported in this study. (TXT)
