# Supplementary material for: Modeling predictors of incomplete antenatal care utilization among reproductive-age women in Ethiopia using machine learning algorithms and SHAP interpretation
Source: PLOS Digit Health. 2026 Jul 9;5(7):e0001489. doi: 10.1371/journal.pdig.0001489 (PMC13349117; doi:10.1371/journal.pdig.0001489)
Supplement: Attachment — Submitted filename: Point by point Response.pdf [file pdig.0001489.s004.pdf]

Date: September, 2025

<u>Dear editorial board member (s) of PLOS Digital Health Journal</u>

The authors have been recalled to revise the manuscript entitled **“Modeling Predictors of Incomplete Antenatal Care Utilization among Reproductive-Age Women in Ethiopia Using Machine Learning Algorithms and SHAP Global and Local Interpretation: Evidence from the 2019 Ethiopian Mini Demographic and Health Survey",** and identified by Submission ID PDIG-D-25-00144 under PLOS Digital Health Journal, which was submitted for publication. So, we received the reviewers and editors revision comments for the improvement of the manuscript before it can be precede to peer-review.

Thank you for receiving comments, suggestions, directions, and questions from the editor and reviewer. As we said usually, we are very happy in receiving constructive and valuable comments for making a better manuscript. Accordingly, we have considered all the comments, questions, directions, and suggestions and provided a point-by-point response letter.

Finally, we have submitted all the required documents in their revised form. We hope that we have addressed all the suggestions, directions, and raised questions and if you believe that point(s) is not addressed, please let us know.

Thank you very much to editor (s), and reviewer

On the behalf of the authors

Yours sincerely,

Correspondence author

Point-by-point response letter

Editor comments

1. Please provide separate figure files in .tif or .eps format.

Author Response: Thank you very much for the comment; we attached the separate files of figures in .tif format in our revised submission

2. In the online submission form, you indicated that [The data used for the study were obtained from the public domain measure DHS website (http://www.measuredhs) upon reasonable request and permission to access the database.]. All PLOS journals now require all data underlying the findings described in their manuscript to be freely available to other researchers, either

a. In a public repository,

b. Within the manuscript itself, or

c. Uploaded as supplementary information.

Author Response: Thank you for the comment; we included the raw data used for this analysis as a supplementary file in the submission system.

Reviewer 1 comments

1. Describe dataset features in more details and its total size and size of (train/test) as a table.

Authors Response : Thank you for this important comment. The detailed description of the total data size, train and test size in line number 45, 269-270 and 372

2. Pseudo code and algorithm steps need to be inserted.

   Authors Response: Thank you for your insightful comment, we revised the methodology section to include detailed description about the modeling steps and the Python code for this specific analysis attached to the submission system as a supplementary file

3. Time spent need to be measured in the experimental results.

   Authors Response: Thank you for important comment, the time period of the study is included in the Study design, setting and period of the methodology section line number 125.

4. Limitation and Discussion Sections need to be inserted.

   Authors Response: Thank you for this interesting comment. We included the discussion and limitation of our main study and comparison with other related studies in this revision. Line number 439-524

5. Comparison with similar studies on a similar dataset need to be inserted (with references).

   Authors Response: Thank you for the comment. Comparison of our findings with other previously done studies is included in the discussion section. Line number 439-524

6. All metrics need to be calculated in the experimental results.

   Authors Response: Thank you for the comment. The performance metrics of all model of under consideration were presented in table 3. Line number 360-361

7. The parameters used for the analysis must be provided in table

   Authors Response: Thank you for the comment. The results of the parameters used in this analysis were presented in table 3 and 4 as well as in Figure 2 – Figure 8

8. The architecture of the proposed model must be provided

   Authors Response: Thank you for the comment. The detailed description about the proposed models was included in the methodology section of the manuscript.

9. Address the accuracy/improvement percentages in the abstract and in the conclusion sections, as well as the significance of these results.

Authors Response: Thank you for the comment. The detailed presentation of the model performance metrics including the accuracy, precision, recall, AUC, and F1 score were presented in the abstract (line number 46 – 49), in the result and conclusion (table 3 and line number, 352 – 357).

10. The authors need to make a clear proofread to avoid grammatical mistakes and typo errors.\

Authors Response: Thank you for your comment. We revised the whole document to edit grammatical mistakes and typo errors in the manuscript.

11. Enhance the clarity of the Figures by improving their resolutions

Author Response: Thank you very much for the comment; we attached the separate files of figures in .tif format to improve the figure resolutions in our revised submission

12. Add future work in last section (conclusion) (if any)

Author Response: Thank you for your comment: We included the conclusion and recommendations based on the key findings of our study; see on line number 545 - 562

13. **The authors need to add recent articles in related work and update them. To improve the Related Work and Introduction sections authors are recommended to review this highly related research work paper:**
    a) Utilizing machine learning to analyze trunk movement patterns in women with postpartum low back pain
    b) Secure and Transparent Lung and Colon Cancer Classification Using Block chain and Microsoft Azure
    a) The power of deep learning in simplifying feature selection for hepatocellular carcinoma: a review
    b) Feature reduction for hepatocellular carcinoma prediction using machine learning algorithms
    c) Machine learning insights into scapular stabilization for alleviating shoulder pain in college students
    d) Employing machine learning for enhanced abdominal fat prediction in cavitation post-treatment

e) Optimizing epileptic seizure recognition performance with feature scaling and dropout layers

f) Predicting female pelvic tilt and lumbar angle using machine learning in case of urinary incontinence and sexual dysfunction

g) Utilizing convolutional neural networks to classify monkey pox skin lesions

h) Revolutionizing core muscle analysis in female sexual dysfunction based on machine learning

i) An analysis of hepatitis C virus prediction using different data mining techniques

j) Optimizing classification of diseases through language model analysis of symptoms

Author Response: Thank you for your comment; we included the findings of the studies related with maternal health issues from above articles and other studies in the introduction (line number 110-128) and in the discussion (line number 468 - 540) sections of the manuscript

Reviewer 2 comments

1. Title and Abstract

- The title is comprehensive but slightly long. Consider shortening it without losing meaning, perhaps by focusing on the method or outcome.

Author Response: Thank you for your important suggestion; we revised the title to shorten and make clear focusing on the problem and the method (line number 1 - 3)

- In the abstract (Lines 26–60), the authors summarize the problem and approach well, but some phrases could be clearer. For instance, “model incomplete ANC utilization” could be revised to “predict incomplete ANC utilization” for precision.

Author Response: Thank you for your insightful comment; we revised the abstract section to improve clarity and consistence (line number 32-33)

- The conclusion in the abstract could benefit from a stronger emphasis on the implications of the findings.

Author Response: Thank you for your important comment; we revised the conclusion section of the abstract to show the implication of the findings (line number 54 - 59)

2. Introduction

Strengths:

- The introduction provides a strong justification for the study.
- Good use of statistics and references to demonstrate the scale of the problem.

Suggestions:

- In Line 68, consider rewriting “Ethiopia is one of eight in SSA” to “Ethiopia is among eight sub-Saharan African countries.”

Author Response: Thank you for your insightful comment; we revised the statement based on your suggestion (line number 69 - 70)

- Line 77 refers to “four prenatal visits” which have now been revised to eight. You could make this clearer by citing the updated WHO guideline directly.

Author Response: Thank you for your important comment; we revised the statement to improve the clarity of the sentence and included the direct citation of the WHO guideline based on your suggestion (line number 79 - 82)

3. Methods

Strengths:

- Detailed explanation of model selection and data pre-processing.
- Application of SMOTE for class imbalance and use of multiple classifiers is commendable.
- SHAP values for interpretability is a modern and well-suited choice.

Suggestions:

- In Line 165, it would be helpful to briefly justify why Yufeng Guo's 7-step ML framework was selected.

Author Response: Thank you for your insightful comment; we included the detailed justification for the use of Yufeng Guo's 7-step ML in our study (line number 200 - 203)

- Line 175 refers to the use of k-nearest neighbour imputation. Please justify why this was preferred over other imputation methods, given the potential for bias in high-dimensional data.

Author Response: Thank you for your important comment; we included the detailed justification for the application of k-nearest neighbor imputation in our study (line number 216 - 219)

- Lines 177–188 on feature engineering are technically correct but could be more reader-friendly. Consider simplifying explanations, especially for non-specialist readers.

Author Response: Thank you for your important comment; we revised the feature engineering section to improve the clarity and easy understanding for all readers (line number 221 - 226)

4. Results

Strengths:

- The results are well presented with appropriate performance metrics for each model.
- Clear reporting of SHAP-based interpretations (global and local).

Suggestions:

- Table 3 (Line 331) contains valuable comparative metrics. However, consider adding statistical significance testing between models.

Author Response: Thank you for your insightful comment; we revised the table of model matrices (table 3) including the statistical significance statics of each model and matrices

- In Lines 357–376, where global SHAP importance is discussed, please explain why certain features (e.g., region or education) may have a higher impact in the Ethiopian context.

Author Response: Thank you for your insightful comment; we included the explanation and justification behind importance/significance of this features in the discussion section compering with already existing findings (483 - 542).

- Consider expanding on the limitations of the SHAP explanation (e.g., sensitivity to correlated variables).

Author Response: Thank you for your important suggestion; we included the limitation of using SHP based model explanation in the limitation and strength of the study (line number 546 - 549)

5. Discussion

Strengths:

- The discussion section draws effectively on previous studies and puts findings into a broader context.
- Regional and sociodemographic disparities are explored in depth.

Suggestions:

- Line 452–454: While regional disparities are acknowledged, a brief mention of ethnic and cultural diversity and how it may influence ANC use would enrich the interpretation.

Author Response: Thank you for your insightful suggestion; we briefly discussed the impact of ethic and cultural diversity on the use of maternal health service and the need for tailored policy and guideline to improve the service in this areas (line number 499 - 510)

- Lines 477–490: The impact of education is well stated, but please mentions how this could be integrated into policy interventions.

Author Response: Thank you for your insightful suggestion; we included brief discussion and suggestion how this could be integrated into policy interventions (line number 529 - 537)

6. Strengths and Limitations

Strengths:

- Acknowledges the black-box nature of ML and the use of SHAP to address it.

Suggestions:

- In Line 519, add more detail on which “significant variables” might have been omitted from the secondary data.

Author Response: Thank you for your important suggestion; we included more variable missed by this study due to the use of secondary data (line number 561 - 563)

7. Conclusion and Recommendations

Strengths:

- The conclusion summarizes key findings and emphasizes action points well.

Suggestions:

- Line 537: "Special consideration to women who live in rural areas…" This could be made more actionable. For instance, recommend "community outreach programs, mobile health services, or targeted subsidies."

Author Response: Thank you for your constructive comments; revised the sentence based on your insightful suggestion (line number 590 -594)

8. Technical and Formatting Issues

- The manuscript contains several overly long paragraphs. Consider splitting them for readability.

Author Response: Thank you for your important comments; we revised the whole manuscript to improve the writing quality and address grammatical problems

- Minor grammatical errors and awkward phrases appear throughout (e.g., "did not have a reach/highest wealth index" in Line 403). A thorough language edit is recommended.

Author Response: Thank you for your important comments; we revised this specific sentence and the whole manuscript to improve the writing quality and address grammatical problems

Recommendation:

This is a relevant and well-executed study that makes an important contribution. However, the manuscript will benefit from:

- Clearer language and structure in some sections,

Author Response: Thank you for your important suggestion; we revised the whole manuscript to improve the writing quality and address grammatical problems

- More concise and action-driven conclusions,
- **Author Response**: Thank you for your important comments; we revised the conclusion section focusing on the clarity, conciseness and driving action-driven points to improve the ANC utilization problem in Ethiopia
- Further justification of methodological choices, and

Author Response: Thank you for your important comments; we included clear and detailed justification for the use of machine learning algorithm predict ANC utilization in Ethiopia

- A light editorial review to polish grammar and clarity.

Author Response: Thank you for your insightful suggestion; we revised the whole manuscript to improve the writing quality, consistence and address grammatical problems
