# Supplementary material for: Modeling predictors of incomplete antenatal care utilization among reproductive-age women in Ethiopia using machine learning algorithms and SHAP interpretation
Source: PLOS Digit Health. 2026 Jul 9;5(7):e0001489. doi: 10.1371/journal.pdig.0001489 (PMC13349117; doi:10.1371/journal.pdig.0001489)
Supplement: Attachment — Submitted filename: Response to Reviewers.pdf [file pdig.0001489.s005.pdf]

Date: February, 2025

<u>Dear editorial board member (s) of PLOS Digital Health Journal</u>

The authors have been recalled to revise the manuscript entitled **“Modeling Predictors of Incomplete Antenatal Care Utilization among Reproductive-Age Women in Ethiopia Using Machine Learning Algorithms and SHAP Interpretation",** and identified by Submission ID PDIG-D-25-00144 under PLOS Digital Health Journal, which was submitted for publication. So, we received the reviewers and editors revision comments for the improvement of the manuscript before it can be precede to peer-review.

Thank you for receiving comments, suggestions, directions, and questions from the editor and reviewer. As we said usually, we are very happy in receiving constructive and valuable comments for making a better manuscript. Accordingly, we have considered all the comments, questions, directions, and suggestions and provided a point-by-point response letter.

Finally, we have submitted all the required documents in their revised form. We hope that we have addressed all the suggestions, directions, and raised questions and if you believe that point(s) is not addressed, please let us know.

Thank you very much to editor (s), and reviewer

On the behalf of the authors

Yours sincerely,

Correspondence author

Reviewer 3 comments

1. The manuscript highlights regional differences, but it does not explain why certain areas, like Somali and Harari, are lagging behind in detail.

Authors response: Thank you very much for your constructive comment we have included the possible explanation for this disparity in the discussion section of the manuscript (See page number 20-21, line number 509- 525).

2. The document explains the choice of specific machine learning (ML) frameworks, like Yufeng Guo's 7-step process, and imputation methods such as KNN. However, it would be stronger if it connected these choices more clearly to the unique features of the Ethiopian Mini Demographic and Health Survey (EMDHS) dataset.

Authors response: Thank you for this valuable comment. We have revised the manuscript to more clearly link the choice of the ML framework and KNN imputation to the specific design and structural features of the EMDHS dataset. (See pages 7–8, Lines 198–213).

3. The study does not consider clinical or health-facility-level factors that often significantly affect antenatal care (ANC) completion.

Authors response: As clearly indicated in the limitations section of the manuscript, the lack of important clinical, health-facility-level, partner related data is one of the most important limitation of the study (See page number: 22 - 23, line number: 570 - 573)

4. In the Methodology section, particularly in the part about "Handling Imbalanced Data. It is suggested to refer from the paper: “AI-Powered Security for IoT Ecosystems: A Hybrid Deep Learning Approach to Anomaly Detection “. The manuscript uses SMOTE for data balancing. This recommended reference discusses the use of hybrid deep learning and generative adversarial networks (GANs) to create synthetic samples, highlighting the importance of balancing in developing robust models for sensitive health data.

Authors response: Thank you for this insightful suggestion; The use of SMOTE over hybrid deep learning and generative adversarial networks (GANs)was justified for its ability to generates reasonable and representative synthetic data for a moderately-sized dataset and does

not distort the underlying relationships in the data. Since the EMDHS dataset contains structured, tabular survey data with moderate sample size and is primarily comprised of categorical variables, there are limitations to the use of GANs as a data augmentation technique since they tend to produce unstable and unrealistic synthetic samples. Conversely, SMOTE works well with structured data by producing synthetic samples through interpolation within local regions in order to preserve the original distribution of the data, thereby broadening the representation of minority classes, while maintaining the interpretability of the data for transparency and reproducibility in the field of public health. The computational requirements associated with developing GANs will be much higher, parameter tunning to obtain an appropriate synthetic data set will be much greater, and any artifacts resulting from the creation of synthetic samples using the GAN approach would potentially invalidate the analysis. Overall, SMOTE provides a simpler, more reliable, and statistically valid option for augmenting the EMDHS based on the structure of the data and the objectives of the study.

5. It would help to provide a clearer explanation for using KNN imputation over other methods, particularly addressing its effectiveness in a dataset with 8% of the data missing completely at random (MCAR).

Authors response: Thank you for this constructive comment. We have revised the manuscript to provide a clearer justification for the use of KNN imputation, particularly in the context of 8% MCAR missingness. The clarification has been incorporated on page number: 8-9, lines number 227 - 235 of the revised manuscript.

6. The discussion on Somali and Harari regions should include factors such as the preference for female healthcare providers and how mobile pastoralist lifestyles affect service continuity.

Authors response: Thank you your constructive suggestion. We have included the preference for female healthcare providers and how mobile pastoralist lifestyles affect service continuity as the possible explanation for this disparity in the discussion section of the manuscript manuscript (See page number 20-21, line number 509- 525).

7. A specific subsection regarding the limitations of SHAP values should be added. This section should explain how SHAP values can be sensitive to correlated variables, leading to inaccurate feature importance.

Authors response: Thank you for this important suggestion. We have included detailed information on the limitation with the use of SHAP based explanation including be sensitive to correlated variables, computational intractability of Shapley-value-based feature importance methods and reflection of internal model biases rather than true feature relevance, and a need to use complex workarounds like causal reasoning in the limitation section of the manuscript. (See page number 22-23, line number 570 – 573)

8. The policy suggestions should be clearer. Instead of saying "special consideration," the manuscript should recommend specific actions, such as "mobile health clinics" or "targeted financial subsidies," for rural women.

Authors response: Thank you for this important suggestion. We have revised the recommendation section improve clarity and specificity (See page number 24, line number 603 - 608)
