# Supplementary material for: Modeling predictors of incomplete antenatal care utilization among reproductive-age women in Ethiopia using machine learning algorithms and SHAP interpretation
Source: PLOS Digit Health. 2026 Jul 9;5(7):e0001489. doi: 10.1371/journal.pdig.0001489 (PMC13349117; doi:10.1371/journal.pdig.0001489)
Supplement: Attachment — Submitted filename: Reviewer Comments.docx [file pdig.0001489.s002.docx]

**Modeling Predictors of Incomplete Antenatal Care Utilization among Reproductive-Age Women in Ethiopia using Machine Learning Algorithms and SHAP Global and Local Interpretation.**

**Review Summary:**

This manuscript addresses an important public health issue in Ethiopia by applying machine learning (ML) algorithms to model and predict incomplete antenatal care (ANC) utilization among reproductive-age women. The use of explainable ML methods, particularly SHAP (Shapley Additive Explanations), enhances the interpretability of the models and provides insightful policy recommendations. The paper is timely, relevant, and technically sound, with a clear methodology and well-structured presentation.

However, some areas require revision and refinement for clarity, coherence, and scientific rigor.

**1. Title and Abstract**

- The title is comprehensive but slightly long. Consider shortening it without losing meaning, perhaps by focusing on the method or outcome.
- In the abstract (Lines 26–60), the authors summarize the problem and approach well, but some phrases could be clearer. For instance, “model incomplete ANC utilization” could be revised to “predict incomplete ANC utilization” for precision.
- The conclusion in the abstract could benefit from a stronger emphasis on the implications of the findings.

**2. Introduction**

**Strengths:**

- The introduction provides a strong justification for the study.
- Good use of statistics and references to demonstrate the scale of the problem.

**Suggestions:**

- In Line 68, consider rewriting “Ethiopia is one of eight in SSA” to “Ethiopia is among eight sub-Saharan African countries.”
- Line 77 refers to “four prenatal visits” which have now been revised to eight. You could make this clearer by citing the updated WHO guideline directly.

**3. Methods**

**Strengths:**

- Detailed explanation of model selection and data preprocessing.
- Application of SMOTE for class imbalance and use of multiple classifiers is commendable.
- SHAP values for interpretability is a modern and well-suited choice.

**Suggestions:**

- In Line 165, it would be helpful to briefly justify why Yufeng Guo's 7-step ML framework was selected.
- Line 175 refers to the use of k-nearest neighbor imputation. Please justify why this was preferred over other imputation methods, given the potential for bias in high-dimensional data.
- Lines 177–188 on feature engineering are technically correct but could be more reader-friendly. Consider simplifying explanations, especially for non-specialist readers.

**4. Results**

**Strengths:**

- The results are well presented with appropriate performance metrics for each model.
- Clear reporting of SHAP-based interpretations (global and local).

**Suggestions:**

- Table 3 (Line 331) contains valuable comparative metrics. However, consider adding statistical significance testing between models.
- In Lines 357–376, where global SHAP importance is discussed, please explain why certain features (e.g., region or education) may have a higher impact in the Ethiopian context.
- Consider expanding on the limitations of the SHAP explanation (e.g., sensitivity to correlated variables).

**5. Discussion**

**Strengths:**

- The discussion section draws effectively on previous studies and puts findings into a broader context.
- Regional and sociodemographic disparities are explored in depth.

**Suggestions:**

- Line 452–454: While regional disparities are acknowledged, a brief mention of ethnic and cultural diversity and how it may influence ANC use would enrich the interpretation.
- Lines 477–490: The impact of education is well stated, but please mention how this could be integrated into policy interventions.

**6. Strengths and Limitations**

**Strengths:**

- Acknowledges the black-box nature of ML and the use of SHAP to address it.

**Suggestions:**

- In Line 519, add more detail on which “significant variables” might have been omitted from the secondary data.

**7. Conclusion and Recommendations**

**Strengths:**

- The conclusion summarizes key findings and emphasizes action points well.

**Suggestions:**

- Line 537: “Special consideration to women who live in rural areas…” This could be made more actionable. For instance, recommend “community outreach programs, mobile health services, or targeted subsidies.”

**8. Technical and Formatting Issues**

- The manuscript contains several overly long paragraphs. Consider splitting them for readability.
- Minor grammatical errors and awkward phrases appear throughout (e.g., “did not have a reach/highest wealth index” in Line 403). A thorough language edit is recommended.

**Recommendation:**

This is a relevant and well-executed study that makes an important contribution. However, the manuscript will benefit from:

- Clearer language and structure in some sections,
- More concise and action-driven conclusions,
- Further justification of methodological choices, and
- A light editorial review to polish grammar and clarity.
